# Supplementary material for: Face, content, criterion and construct validity assessment of a newly developed tool to assess and classify work–related stress (TAWS– 16)
Source: PLoS One. 2023 Jan 6;18(1):e0280189. doi: 10.1371/journal.pone.0280189 (PMC9821462; doi:10.1371/journal.pone.0280189)
Supplement: S1 Table — (DOCX) [file pone.0280189.s001.docx]

**S1 Table. Rating of Face Validity of items of TAWS – 16 by experts**

| **SL No** | **Description of Items** | **Average Scores** | | | |
| --- | --- | --- | --- | --- | --- |
|  |  | **Level of Understanding** | **Unambiguity** | **Clarity** | **Less Chance of Misinterpretation** |
|  |  | Mean±SD | Mean±SD | Mean±SD | Mean±SD |
| ST1 | I experience time/deadline pressures due to heavy workload | 8.7±1.92 | 8.7±1.09 | 8.7±1.48 | 9.1±1.05 |
| ST2 | I am required to multitask regularly and perform different roles, often leaving me unclear of my exact role in organization | 8.5±1.23 | 8.2±1.56 | 8.2±1.30 | 8±2.39 |
| ST3 | Multiple demands are placed on me by different superiors in the organization | 8±1.73 | 7.2±2.58 | 8.4±1.33 | 7.2±2.58 |
| ST4 | I do not receive the respect and recognition at work I deserve from my colleagues and seniors | 9±0.86 | 8.6±1.93 | 8.8±1.36 | 8.4±2.55 |
| ST5 | I have not been promoted regularly as per my performance or company rules | 8.4±1.33 | 8.3±1.58 | 8.3±1.41 | 7.3±3.16 |
| ST6 | I am having a feeling of lack of job security in current job | 8.5±1.87 | 8.3±1.80 | 8.5±1.59 | 7.7±1.85 |
| ST7 | My efforts are not adequately remunerated or rewarded by the organization | 8.6±1.32 | 8.6±1.11 | 8.6±1.32 | 8.1±1.61 |
| ST8 | I am required to work longer than usual working hours | 9.3±0.70 | 8.6±1.5 | 9±0.86 | 7.8±3.05 |
| ST9 | The organization/industry/senior management does not involve me/other employees in making decisions about workload, working style etc. | 7.7±1.98 | 7.8±2.14 | 8.2±1.98 | 7.1±2.97 |
| ST10 | There is a friction or non-cordial relationship between colleagues or superiors | 8.4±1.23 | 8.5±1.13 | 8.2±1.39 | 7.3±2.73 |
| ST11 | I am finding difficulty in getting work done or delegating responsibilities to sub ordinates/colleagues | 8.7±1.20 | 8.6±1.73 | 8.5±1.81 | 8.5±1.74 |
| ST12 | Working conditions are not comfortable in terms of space, ventilation, lighting, equipment | 8.7±1.56 | 8.7±1.56 | 8.6±1.73 | 8.5±1.94 |
| ST13 | I am not given supportive and adequate feedback/ appraisal on the work I do | 8.6±1.5 | 8.6±1.5 | 9.1±0.92 | 7.6±2.87 |
| ST14 | I do not get help and support I need from colleagues/seniors/ supervisors | 9±0.86 | 8.4±2.18 | 9.1±0.78 | 7.5±2.96 |
| ST15 | I am not trained regularly & adequately by the organization to improve our knowledge & skills to work | 8.8±1.05 | 8.5±1.74 | 8.7±1.30 | 8.4±1.66 |
| ST16 | I am finding it difficult to balance work and home life | 9.1±1.05 | 9.1±0.78 | 9.3±0.70 | 9.2±0.83 |
|  | Overall Average Scores | 8.62±0.39 | 8.41±0.42 | 8.64±0.34 | 7.98±0.65 |
