# Supplementary material for: Face, content, criterion and construct validity assessment of a newly developed tool to assess and classify work–related stress (TAWS– 16)
Source: PLoS One. 2023 Jan 6;18(1):e0280189. doi: 10.1371/journal.pone.0280189 (PMC9821462; doi:10.1371/journal.pone.0280189)
Supplement: S2 Table — (DOCX) [file pone.0280189.s002.docx]

**S2 Table. Rating and Calculation of I-CVI and S-CVI of Work Related Factors and Symptoms Suggestive of Work Stress for TAWS – 16**

Total Items = 32

Number of Experts= 8

| **SL No** | **Description of Items** | **Rating 3 and 4** | **Rating 1 and 2** | **I-CVI** | **Modified Kappa** |
| --- | --- | --- | --- | --- | --- |
| ST1 | I experience time/deadline pressures due to heavy workload | 8 | 0 | 1 | 1 |
| ST2 | I am required to multitask regularly and perform different roles, often leaving me unclear of my exact role in organization | 8 | 0 | 1 | 1 |
| ST3 | Multiple demands are placed on me by different superiors in the organization | 7 | 1 | 0.857 | 0.852 |
| ST4 | I do not receive the respect and recognition at work I deserve from my colleagues and seniors | 7 | 1 | 0.857 | 0.852 |
| ST5 | I have not been promoted regularly as per my performance or company rules | 7 | 1 | 0.857 | 0.852 |
| ST6 | I am having a feeling of lack of job security in current job | 7 | 1 | 0.857 | 0.852 |
| ST7 | My efforts are not adequately remunerated or rewarded by the organization | 6 | 2 | 0.75 | 0.719 |
| ST8 | I am required to work longer than usual working hours | 7 | 1 | 0.857 | 0.852 |
| ST9 | The organization/industry/ senior management does not involve me/other employees in making decisions about workload, working style etc | 5 | 3 | 0.625 | 0.52 |
| ST10 | There is a friction or non-cordial relationship between colleagues or superiors | 8 | 0 | 1 | 1 |
| ST11 | I am finding difficulty in getting work done or delegating responsibilities to sub ordinates/colleagues | 5 | 3 | 0.625 | 0.52 |
| ST12 | Working conditions are not comfortable in terms of space, ventilation, lighting, equipment | 4 | 4 | 0.5 | 0.311 |
| ST13 | I am not given supportive and adequate feedback/ appraisal on the work I do | 6 | 2 | 0.75 | 0.719 |
| ST14 | I do not get help and support I need from colleagues/seniors/supervisors | 7 | 1 | 0.857 | 0.852 |
| ST15 | I am not trained regularly & adequately by the organization to improve my knowledge & skills to work | 3 | 5 | 0.375 | 0.2 |
| ST16 | I am finding it difficult to balance work and home life | 8 | 0 | 1 | 1 |
| SY1 | Excessive tiredness | 8 | 0 | 1 | 1 |
| SY2 | Lack of energy | 8 | 0 | 1 | 1 |
| SY3 | Lack of motivation | 7 | 1 | 0.857 | 0.852 |
| SY4 | Tendency to make errors at work | 6 | 2 | 0.75 | 0.719 |
| SY5 | Increased irritability | 7 | 1 | 0.857 | 0.852 |
| SY6 | Frequent fights with colleagues | 7 | 1 | 0.857 | 0.852 |
| SY7 | Increased absence from work | 6 | 2 | 0.75 | 0.719 |
| SY8 | Disturbed sleep | 8 | 0 | 1 | 1 |
| SY9 | Headache | 8 | 0 | 1 | 1 |
| SY10 | Backaches | 8 | 0 | 1 | 1 |
| SY11 | Smoking | 8 | 0 | 1 | 1 |
| SY12 | Alcohol use | 6 | 2 | 0.75 | 0.719 |
| SY13 | Skin problems | 4 | 4 | 0.5 | 0.311 |
| SY14 | Gastric / digestion problems | 7 | 1 | 0.857 | 0.852 |
| SY15 | Increase in heartbeat | 7 | 1 | 0.857 | 0.852 |
| SY16 | Increased appetite | 6 | 2 | 0.75 | 0.719 |

**S-CVI/Average**= Average of I-CVI= **0.829**
