# Supplementary material for: Face, content, criterion and construct validity assessment of a newly developed tool to assess and classify work–related stress (TAWS– 16)
Source: PLoS One. 2023 Jan 6;18(1):e0280189. doi: 10.1371/journal.pone.0280189 (PMC9821462; doi:10.1371/journal.pone.0280189)
Supplement: Attachment — Submitted filename: Response to Reviewers.docx [file pone.0280189.s003.docx]

[PONE-D-22-20570] - [EMID:dd7076c02d717c61]

**Response to comments by reviewer**

|  | **Comments to author by reviewer** | **Response to reviewer** |
| --- | --- | --- |
| 1. Is the manuscript technically sound, and do the data support the conclusions? | Yes | We thank the reviewer for this comment |
| 2. Has the statistical analysis been performed appropriately and rigorously? | Yes | We thank the reviewer for this comment |
| 3. Have the authors made all data underlying the findings in their manuscript fully available? | Yes | We thank the reviewer for this comment |
| 4. Is the manuscript presented in an intelligible fashion and written in standard English? | No | The manuscript has been revised to correct language, sentence, and grammar. Changes are reflected in Track mode |
| 5. Review Comments to the Author |  |  |
| Reviewer 1 | 1- please report the C VR for content validity | CVI and Modified Kappa are reported as measure of content validity in our study as CVI and Kappa are reported as better and commonly used measure of content validity [1]. We reported Modified Kappa to eliminate chance agreement  Based on the study by Ayre and Scally [2], the critical value of CVR for 8 experts would be 0.75. Our experts ranged from diverse occupational health backgrounds (occupational health experts, public health experts, psychiatrists, psychologists, IT professionals) due to which CVR critical value was difficult to achieve for items which were otherwise found appropriate with CVI and Modified Kappa. Hence, we did not use CVR as a measure of content validity in our analysis |
| Reviewer 1 | For data collection(items) what do you do? interview? in which method? qualitative research in which method? is it mixed method? this part is ambiguous | Thank you for the comment  For face validity and content validity – Hard copy of the study instrument was personally handed over to the experts and context was explained to them. For experts staying outside Bengaluru city, the form was emailed, and context was explained over telephone.  The experts filled the form and provided it back to the investigator  For assessment of criterion and construct validity, the investigator made a phone call to random IT employees and sought verbal consent for participation.  For employees who were willing to participate, a web-link to TAWS-16 was emailed to them. The participating employees created a login and password and provided informed consent before filling out the questionnaire in email (self-reporting).  No qualitative research techniques were employed in this study.  Same is mentioned in the materials and methods |
| Reviewer 1 | likelihood is better than PCA please explain why PCA selected | Thank you for the comment.  We used EFA and likelihood for construct validity analysis. The same is mentioned in the materials and methods.  PCA was not done. |
| Reviewer 1 | Table 2 can be eliminated and the data report in the text | Thank you for the comment  The previous has been eliminated and data reported in the text and renamed |

Reference

1.	Wynd CA, Schmidt B, Schaefer MA. Two quantitative approaches for estimating content validity. Vol. 25, Western Journal of Nursing Research. 2003. p. 508–18.

2.	Ayre C, Scally A. Critical Values for Lawshe’s Content Validity Ratio. Meas Eval Couns Dev. 2013 Dec 13;47:79–86.
